# Cone Beam Micro-CT System for Small Animal Imaging and Performance Evaluation

**DOI:** 10.1155/2009/960573

**Published:** 2009-09-22

**Authors:** Shouping Zhu, Jie Tian, Guorui Yan, Chenghu Qin, Jinchao Feng

**Affiliations:** ^1^Medical Image Processing Group, Institute of Automation, Chinese Academy of Sciences, Beijing 100190, China; ^2^College of Electronic Information & Control Engineering, Beijing University of Technology, Beijing 100124, China

## Abstract

A prototype cone-beam micro-CT system for small animal imaging has been developed by our group recently, which consists of a microfocus X-ray source, a three-dimensional programmable stage with object holder, and a flat-panel X-ray detector. It has a large field of view (FOV), which can acquire the whole body imaging of a normal-size mouse in a single scan which usually takes about several minutes or tens of minutes. FDK method is adopted for 3D reconstruction with Graphics Processing Unit (GPU) acceleration. In order to reconstruct images with high spatial resolution and low artifacts, raw data preprocessing and geometry calibration are implemented before reconstruction. A method which utilizes a wire phantom to estimate the residual horizontal offset of the detector is proposed, and 1D point spread function is used to assess the performance of geometric calibration quantitatively. System spatial resolution, image uniformity and noise, and low contrast resolution have been studied. Mouse images with and without contrast agent are illuminated in this paper. Experimental results show that the system is suitable for small animal imaging and is adequate to provide high-resolution anatomic information for bioluminescence tomography to build a dual modality system.

## 1. Introduction

Dramatic advances in imaging technology, especially for small animal imaging, have been an important driving force in establishing the field of molecular and genomic imaging [1]. Micro-CT system for small animal imaging has been studied since 1990s [2–7] and has played a critical role in the evolution of molecular imaging [8, 9].It can obtain high-resolution anatomic information and can be combined with other modalities [10, 11], such as nuclear imaging [12, 13] and optical imaging [14]. 

A prototype cone-beam micro-CT system for small animal imaging has been developed by our group. In this system, we use a microfocus X-ray source and a complementary metal oxide semiconductor (CMOS) based flat-panel detector. FDK [15] method is adopted for 3D reconstruction. In order to accelerate CT reconstruction speed, we develop reconstruction software using Graphics Processing Unit (GPU) hardware [16]. 

The aim for building such a system is to develop a dual modality system integrated with a micro-CT scanner and a bioluminescence tomography (BLT) system. The CT system is designed to provide anatomic information for BLT. However, the spatial resolution of BLT is about several millimeters or submillimeters, so the anatomic structure with less than 100 micron is enough. Moreover, main organs, such as lung, bone, kidney, and liver, should be discriminated in CT image. 

In this paper, the prototype micro-CT system is introduced, including overview of the system, geometric misalignment calibration, GPU-based image reconstruction and post-processing software. System performances have been evaluated in terms of spatial resolution, image uniformity and noise, and low contrast resolution. Furthermore, some mouse images with and without contrast agent are presented to show the overall performance of the system.

## 2. System Description

### 2.1. Overview of the System

**Figure 1:**
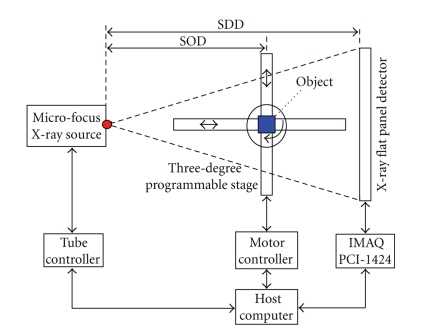
Schematic diagram of the micro-CT system. It consists of a microfocus X-ray source, a flat panel X-ray detector and a three-degree programmable stage. All of them are mounted on an optical bench and controlled by a host computer.

The prototype micro-CT system consists of a microfocus X-ray source, a three-dimensional (x-y translation and rotation) programmable stage with mouse holder and a flat-panel X-ray detector. All of them are mounted on an optical bench in a shielded laboratory and controlled by a host computer. A schematic diagram of the system is shown in Figure 1. The source to detector distance (SDD) and source to object distance (SOD) are variable to change the magnification ratio, cone-beam angle, and field of view (FOV) of the system. In most cases, the SDD is set to 498 mm and the magnification ratio M is set to 1.3, where M = SDD/SOD.

The X-ray source (UltraBright, Oxford Instruments, USA) has a microfocus continuously adjustable from 13 *μ*m to 40 *μ*m and the power density has been rated to 2.5 W/*μ*m. This holds true until it reaches the minimum spot size possible. 2.5 W/*μ*m is not absolute but rather a rule of thumb. The target voltage of the X-ray tube is 20 to 90 kVp, with maximum output power 80 W. The X-ray flat panel detector (C7942CA-02, Hamamatsu, Japan), based on CMOS technology with a column CsI scintillator plate, has a 120 mm × 120 mm photodiode area with 50 *μ*m pixel size. It acquires 12 bits digital images with dynamic range 2000. The image data are transferred to the host computer by a frame grabber card (IMAQ PCI-1424, National Instruments, USA) in the system. 

The three-dimensional programmable stage consists of two motorized translation stages (PSA200-11, Zolix Instruments, China) and one motorized rotation stage (RAK-100, Zolix Instruments, China). The resolution of the rotation stage is up to 0.00125°. The magnification ratio can be changed by adjusting the position of the object (mouse or rat) using the motorized translation stages, which will significantly affect the spatial resolution and the FOV. The FOV is usually large enough to acquire the whole body imaging of a normal size mouse in a single scan with a small magnification ratio.

### 2.2. Geometric Calibration

Precise geometric alignment is crucial to high quality imaging reconstruction in CT system. Several methods [17–19] have been proposed to evaluate and calibrate the geometric misalignment. In our system, the method proposed by Yang [18] is used. As is stated in [18], the rotation axis is defined as the Z axis of the system. The axis which passes through the cone vertex (X-ray tube focal spot) and perpendicular to the Z axis is defined as the X axis. The axis perpendicular to the X-Z plane is defined as the Y axis. There are seven parameters to describe the system geometry:

1. source to detector distance (SDD),
2. source to object distance (SOD),
3. *u*_0_, horizontal location of the intersection of the X axis and detector plane,
4. *v*_0_, vertical location of the intersection of the X axis and detector plane,
5. *η*, in-plane rotation angle, the rotation angle of the detector plane along X axis,
6. *φ*, out-of-plane rotation angle, the rotation angle of the detector plane along the axis of *v* = *v*_0_,
7. *σ*, out-of-plane rotation angle, the rotation angle of the detector plane along the axis of *u* = *u*_0_,

The two out-of-plane rotation angles, *σ* and *φ*, are quite difficult to determine with reasonable accuracy and have only a small influence on image quality, so *σ* and *φ* could be assumed to zero during geometric calibration, and then five other parameters need to be estimated. Therefore, the method with multiple projection images acquired from rotating point-like objects (metal ball bearings) are used to estimate these five parameters [18].

During experiments, it is found that the horizontal location parameter *u*_0_ has a great influence on image quality. Though Yang's method could achieve high accuracy, there still exists a small residual detector horizontal offset sometimes, which may be caused by the projection noise in the geometric evaluation [18]. We denote the residual horizontal offset as Δ*u*, which is the difference between the actual and estimated value of *u*_0_ after Yang's geometric calibration. If the residual offset value is large, it will degrade the reconstruction image, especially when high resolution is desired.

In the developed system, a wire phantom is utilized to assess the geo-calibration result, and a simple method is proposed to evaluate the horizontal offset through the wire phantom reconstruction image. Considering a wire phantom with the wire diameter significantly smaller than the system spatial resolution, cross-sectional view of the phantom reconstruction image can be regarded as the point spread function (PSF) of the system. Through 1D PSF, the profile of the PSF image, one can access geometric calibration effect quantitively. If there is no detector residual horizontal offset, the wire in the cross-sectional view will be a point. While if a small horizontal offset exists after geometric calibration, PSF will be spreaded and its intensity will become smaller. Camparing the peak value of PSF with different geometry calibration, we can determine which one leads to a better calibration result.

If the residual horizontal offset is large enough, a ring will appear in the cross-sectional view instead of a point, and the radius of the ring is related to the horizontal offset value. We have proposed a simple method to assess the horizontal offset, which is derived as follows.

**Figure 2:**
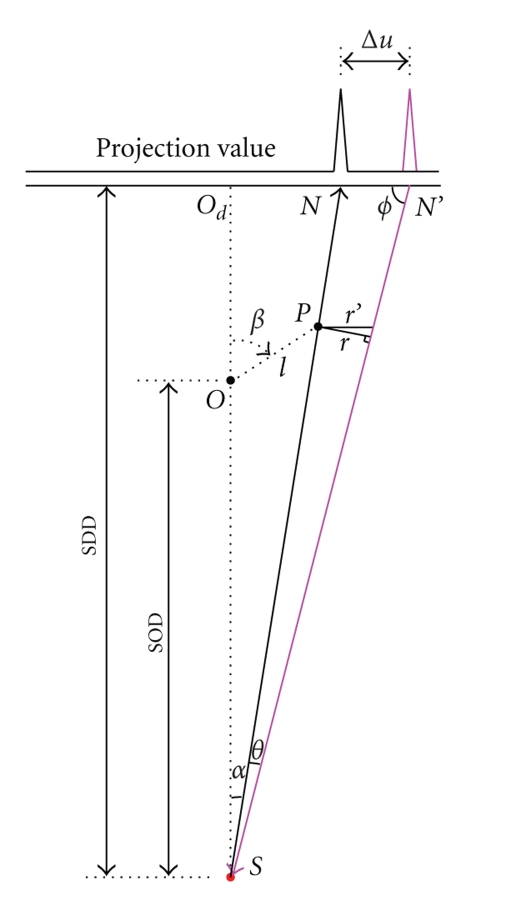
Projection and backprojection of a point with a small detector horizontal offset.

**Figure 3:**
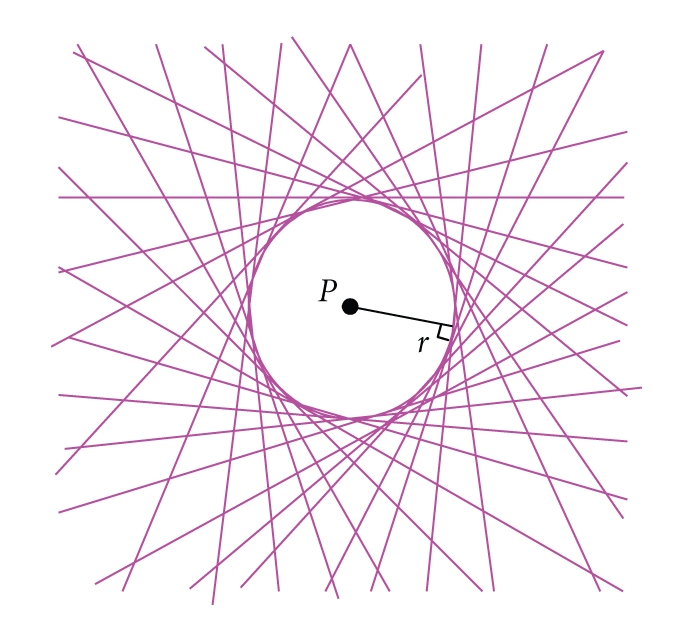
Illumination the production of the ring caused by the horizontal offset.

Let *S*, *O* and *O*_*d*_ be the focal spot of the X-ray source, the center of the rotational stage and the center of the flat panel detector, respectively. We use *P* to denote a point in the tungsten wire near horizontal mid-plane of the cone beam, where the cone angle can be neglected. The distance between the wire and the rotational axis is *l*. Figure 2 shows that the rotational stage turns an angle *β* (0 ≤ *β* < 2*π*), and the projection of the point *P* will be along the radial *SP* or *SN* which makes an angle *α* with the center ray *SO*. Owing to the effect of the horizontal offset Δ*u*, back-projection will start from *N′* and along the radial *N′S* which makes an angle *θ* with *SP*. The distance from point *P* to the line *N′S* is *r*. Figure 3 illuminates that the tail portions of the line are cancelled and a ring with radius *r* around the point *P* is formed when the neighboring views are closely placed. As Δ*u* is very small, the following relationships can be derived:

(1) $$\theta \approx \frac{\Delta u}{\text{SDD}},$$

(2) $$\alpha =\arctan\left(\frac{l\;\sin\;\beta }{\text{SOD}+l\;\cos\;\beta }\right),$$

(3) $$\phi =\frac{\pi }{2}-\alpha -\theta ,$$

(4) $$r^{\prime }=\frac{r}{\sin\;\phi },$$

(5) $$\frac{\Delta u}{r\prime }=\frac{\text{SDD}}{\text{SOD}+l\;\cos\;\;\beta }.$$

Substituting (3) and (4) into (5), we have

(6) $$\frac{\Delta u}{r}=\frac{\text{SDD}}{\left(\text{SOD}+l\;\cos\;\;\beta \right)\sin\left(\pi /2-\alpha -\theta \right)}.$$

As the wire is near the phantom center, the value *l* will be very small, only a few millimeters, when the phantom is put in the center of the rotational stage, thus *l* ≪ SOD. Δ*u* is significantly smaller than SDD, Δ*u* ≪ SDD, then (6) can be simplified to

(7) $$\frac{\Delta u}{r}=\frac{\text{SDD}}{\text{SOD}}=M,$$

where *M* is the magnification ratio of the system. By (7), the horizontal offset can be evaluated and the ring radius value can be measured from the wire reconstruction image. Reconstruction image with smaller voxel may help to detect the ring and determine the radius of the ring. Though the offset value could be calculated, yet the offset direction could not be determined. So the offset needs to be calibrated in both left and right to confirm the correct direction.

It should be noted that the above analysis is an ideal case. In real experiments, the ring may be not very clear because of the effects of noise and wire size. So we have to average many cross-sectional slices to identify the ring clearly. In addition, when the horizontal offset is small, the ring may not appear in the image. In this condition, if one wants to dertermine whether the calibration result is good or not, a small horizontal offset (about a pixel) should be added artificially. Then the offset could be evaluated by the above method. If the evaluted value is equal to the added one, it indicates that the former geometric calibration is good. Otherwise, calibrate the geometry by the new evaluated horizontal offset. 1D PSF can be used to determine the better geometric caliration results.

### 2.3. Data Preprocessing, Image Reconstruction and Postprocessing

Due to dark imaging offset, pixel gain and defective pixels, raw data from X-ray detector show spatial variation, therefore it is necessary to perform data preprocessing before reconstruction, including dark current subtraction, flat field division, and defective pixels interpolation. 

After data pre-processing, FDK method is adopted for 3D reconstruction in the developed system. Because an exact reconstruction is impossible for circular cone-beam scans due to data insufficiency [20, 21], FDK is an approximate algorithm and the reconstruction images using FDK algorithm have artifacts such as low-intensity drop when the cone angle is large. However, our experiments and other literatures [22, 23] have suggested that the reconstruction images are acceptable when the cone angle is relatively small (about less than 10°). It is well known that 3D CT image reconstruction is computationally demanding, so a software using GPU hardware is developed to improve the reconstruction speed [15]. It takes about 5.2 seconds to reconstruct a 512 cubed volume from 360 views of the size of 512 × 512 on a 2.66 GHz dual-core Intel PC with 2 GB RAM hosting a Nvidia Geforce 8800GTX card. Large data reconstruction may need tens of or hundreds of seconds, because data reading from hard disk to RAM costs lots of time.

We use 3D medical image processing and analyzing software 3DMed (v. 2.2.0), developed by the Medcal Image Group, Institute of Automation, Chinese Academy of Sciences (http://www.3dmed.net/, http://www.mitk.net/), to display and post-process the 3D micro-CT data. It is a free software and combines the function of medical image segmentation, registration and visualization. Furthermore, it supports the creation of plug-ins to incorporate new processing algorithms [24].

## 3. System Performance Evaluation

In order to evaluate the system characteristics, the reconstruction images are assessed in terms of modulated transfer function (MTF), uniformity and noise level, and low contrast resolution. The overall performance of the micro-CT system is studied by mouse imaging. Phantoms and mouse scanning protocols are listed detailedly in Table 1.

### 3.1. Modulation Transfer Function of the System

**Figure 4:**
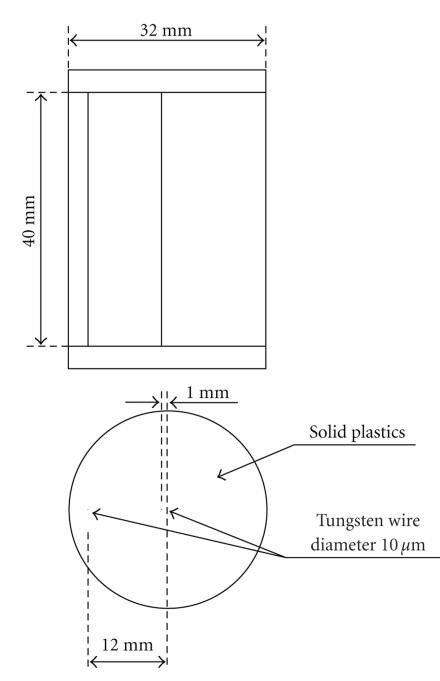
Dimensions of the micro-CT wire phantom.

A wire phantom (Micro-CT wire phantom, QRM, Germany), as shown in Figure 4, is utilized to measure the PSF and MTF of the micro-CT system in our study. The cylinder phantom contains two tungsten wires in solid material aligned parallel to the phantom axis of rotation. One of the wires is slightly off center, the other 12 mm away from the center in order to allow estimating image quality in the periphery.

The wire should be perpendicular to the horizontal plane to measure the MTF. The two tungsten wires in the phantom are designed to parallel to the phantom axis. When the cylinder phantom is put on the rotation stage perpendicularly, the two wires are expected to perpendicular to the horizontal plane. We can judge whether the wire slopes or not by the sagittal and coronal view of the reconstruction image. If there is no slope observed from the sagittal view and coronal view, we assume that the wire is perpendicular to the horizontal plane. Otherwise, replace it and scan again. 

The wire phantom is scanned according to the above protocols to acqure the system modulation transfer function. The reconstruction image of the wire can be regarded as the PSF of the system. Calculating the modulus of the Fourier transform of the PSF and normalizing to 1 at spatial frequency at zero will obtain the MTF. 

Different magnification ratios, 1.3 and 2.36, are set to observe geometric position effect on spatial resolution. As there are two wires in the phantom, one could evaluate the spatial resolution both near the rotation axis and off away from the axis in a single scan measurement. Ramp function and cosine window function (Hamming window) are used respectively as the filter kernel during 3D reconstruction to study the influence of different filter kernels.

### 3.2. Uniformity and Noise in Reconstruction Image

**Figure 5:**
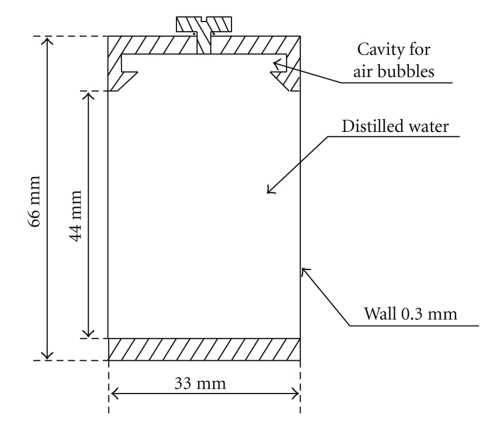
Dimensions of the micro-CT water phantom.

Imaging uniformity and noise of the reconstruction image are investigated with a cylindrical phantom filled with distilled water (Micro-CT water phantom, QRM, Germany), as shown in Figure 5.

The radial profile of the reconstruction image is plotted to illuminate the uniformity qualitatively. A quantitative assessment of the signal variation, from center to periphery, is performed by calculating the mean values and standard deviations in five regions of interest (ROIs), one in the center of phantom and four in the periphery, and the average differences in signal intensity values between the central and the perihperal regions are calculated [25]. The size of ROI is set to 3 × 3 × 3 mm^3^. Five different exposure intensities are used to determine the relationship between exposure and image noise. The tube is set at 50 kVp with the tube current varying from 0.4 mA to 1.2 mA, which corresponds to exposure from 0.24 mAs to 0.72 mAs for a single projection. Beam hardening correction is implemented to reduce artifacts caused by polychromatic X-ray beam [26].

The total measured noise can be considered as a quadrature summation of photon noise and system noise [25], then one can obtain

(8) $$\sigma_{m}^{2}=\frac{A}{E}+\sigma_{s}^{2},$$

where *σ*_*m*_^2^ is the measured noise variance, *σ*_*m*_ is standard deviation. Also, *E* is the exposure for a single view in mAs, and *A* and *σ*_*s*_^2^ are constant terms. In our system, the system noise variance *σ*_*s*_^2^ is significantly small and can be neglected. The constant term *A* is a scaling factor between noise variance and exposure.

### 3.3. Low Contrast Resolution

The low contrast resolution is determined primarily by the noise level in the image. Assume that noise follows Gaussian distribution. If we want to distinguish the low-contrast objects from their background with false positive and false negative rate 5%, the means of the two distributions should be separated by 3.29 *σ*_*m*_ [27]. For 50% false-positive and false-negative rate, 0.83 *σ*_*m*_ separation are needed.

### 3.4. Small Animal Imaging

For *in vivo* small animal imaging, artifacts caused by physiological motion are inevitable. To reduce the motion blurring due to breathing or heart beating, the systems with high framing rate or respiratory gating has been studied [25, 28, 29]. None of these techniques has been implemented in our system and great efforts have been made to reduce motion effects under the current condition. Anesthesia is a key problem. Compared with isoflurane inhalation, intraperitoneal injection of Urethane or pentobarbital sodium may decrease respiratory motion during scanning. Thus we use intraperitoneal injection method to anaesthetize mouse during scanning. In addition, as the purpose of the system is to provide anatomic structure for BLT, which resolution is about several millimetres or submillimetres, so some slight motion artifacts are acceptable.

The overall performance of the micro-CT system is assessed by mouse scanning. The mouse was anesthetized with pentobarbital sodium and no contrast agent was used during scanning. The cross-sectional view, coronal view, sagittal view, and the bone structure of the mouse are illuminated in the following section.

In order to enhance soft tissues contrast resolution, exogenous contrast agent is adopted in another mouse experiments. After fasting for 24 hours, a 20 g BALB/C mouse is slowly injected Fenestra LC (ART, Montreal, Canada) at a dose of 15 mL/kg over a period of 30–60 seconds via the lateral tail vein. It is anesthetized with Urethane and scanned by our micro-CT system at 3.5 hours postinjection. The reconstruction data of the mouse can be free downloaded from http://www.mosetm.net/.

## 4. Results

### 4.1. Horizontal Offset Calibration

The wire phantom shown in Figure 4 is used to assess the geometric calibration results. It is reconstructed with Ram-Lak filter and the voxel size is 0.01 × 0.01 × 0.05 mm^3^. However, 100 slices near the horizontal mid-plane are averaged to reduce the noise effect. 

**Figure 6:**
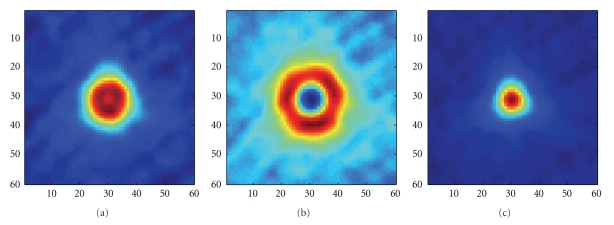
Wire reconstruction results with different horizontal offset calibration: (a) reconstruction image with a horizontal offset, (b) calibration implemented in incorrect direction, (c) excellent geometric calibration results.

Figure 6(a) shows that a ring exists in the cross-sectional view of the wire phantom, which arises from a small horizontal offset after geometric calibration. In Figure 6(a), the radius of the ring is mesured about 0.035 mm. The magnification ratio is 1.3. According to (7), the horizontal offset can be determined, that is, 0.045 mm, about 0.9 pixel. Then we calibrate the offset and reconstruct the wire image once again. Because the misalignment direction is unknown, the calibration have to be implemented in left and right to determine the correct direction. Figure 6(b) shows the calibration result in incorrect direction. Figure 6(c) gives an excellent geometric calibration results, where a point appears in the image instead of a ring. 

**Figure 7:**
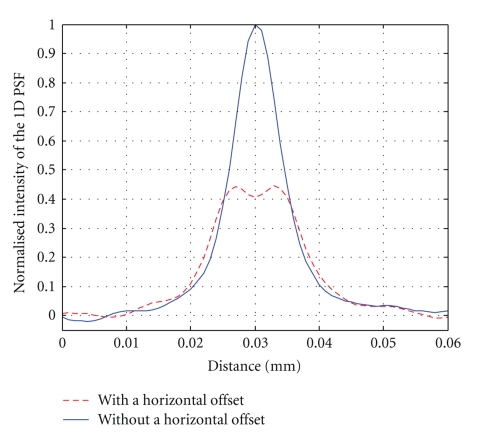
1D point spread function with and without a horizontal offset.

In order to assess the geometric results quantitively, normalized 1D PSFs are ploted in Figure 7, which are corresponding to Figures 6(a)  and 6(c). Due to horizontal geometric offset, the PSF has been spreaded and its peak is decreased and forked. It should be noted that when the horizontal offset is not large, the forked peak will not appear but the peak value will be still lower than the excellent geometric calibration result. Campared the PSF peak values obtained with different horizontal offsets, the optimal geometric configration can be determined.

### 4.2. Modulation Transfer Function of the System

**Figure 8:**
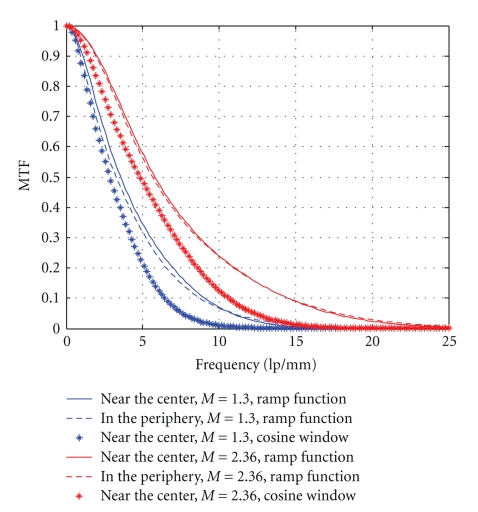
Modulation transfer function (MTF) of the system.

Figure 8 shows the modulation transfer function of the cone-bean micro-CT system. The effects of the magnification ratio and filter kernel are illuminated in the figure clearly. The spatial resolution in the periphery of the phantom is a little lower than that near the center, but not obviously. 

At a lower magnification ratio (*M* = 1.3), the spatial resolution is about 9 lp/mm at 10% of the MTF curve with ramp function kernel and 6.5 lp/mm with cosine window kernel, while it reaches about 14.5 lp/mm with ramp function kernel and 10.5 lp/mm with cosine window kernel at a higher magnification ratio (*M* = 2.36).

### 4.3. Uniformity and Noise in Reconstruction Image

**Figure 9:**
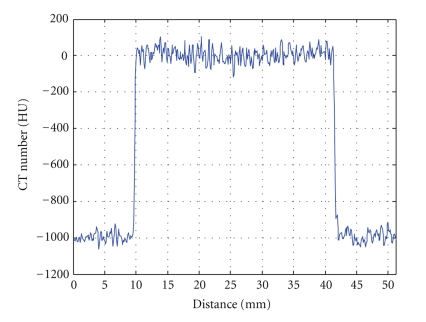
Radial profile of the reconstructed water phantom.

The radial profile of the reconstruction water phantom is plotted in Figure 9 to illuminate the uniformity of the image. Table 2 gives the mean value and standard deviation of the signals in five different ROIs with different exposure intensities. Signal average values acquired in different exposure intensities share almost the same value, while the standard deviation decreases with the increment of the exposure intensity. For all exposure intensities, the differences of the average signal intensities from center to periphery are all less than one standard deviation of the signals. 

**Figure 10:**
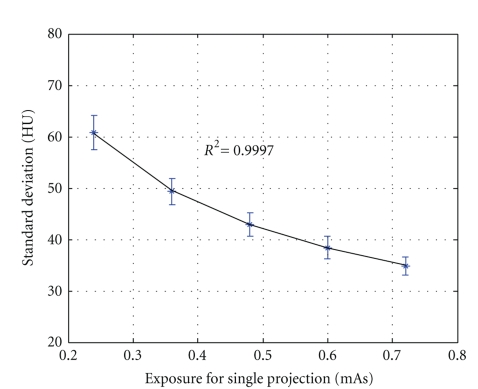
Relationship between voxel noise standard deviation and exposure intensity.

Figure 10 shows the relationship between standard deviation of voxel noise (in HU) and the exposure intensity (in mAs), which has been fitted to (8) and obtains

(9) $$\sigma_{m}^{2}=\frac{883}{E},$$

where the system noise variance *σ*_*s*_^2^ is neglected because it is significant small. Equation (9) indicates that the voxel noise variance is inversely proportional to the exposure intensity.

### 4.4. Low Contrast Resolution

As shown in Figure 10, the standard derivation of the signal noise is about between 35 HU and 60 HU. Take 50 kVp, 1.2 mA for example, the standard derivation is 34.8 HU, which means that 114.5 HU difference is needed to distinguish the low contrast objects from their background with false positive and false negative rate 5%, and 28.9 HU is needed to reach 50% false positive and false negative rate.

### 4.5. Small Animal Imaging

**Figure 11:**
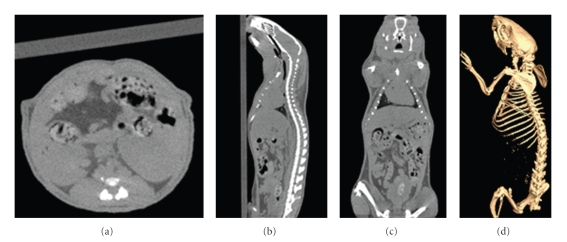
3D reconstruction results of a mouse: (a) transaxial view, (b) sagittal view, (c) coronal view, and (d) bone structure.

Figure 11 shows the reconstruction results of a laboratory mouse without contrast agent. Figures 11(a), 11(b), and 11(c) are the transaxial view, the sagittal view, and the coronal view of the mouse, respectively. Figure 11(d) shows the bone structure of the mouse segmented from the reconstruction data using the software 3DMed.

**Figure 12:**
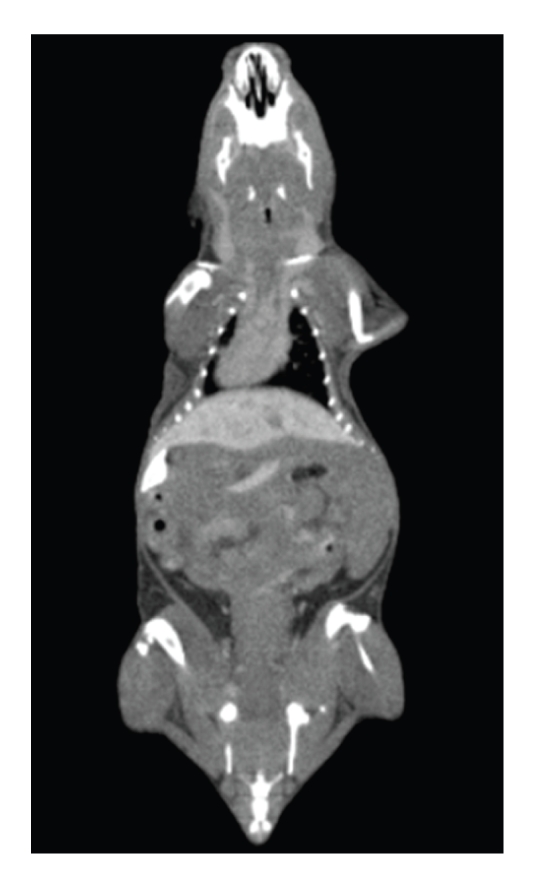
Mouse image enhanced by Fenestra LC.

Delineating the organ of liver from Figure 11 is difficult. While Figure 12 shows excellent liver contrast enhancement which is achieved at 3.5 hours postinjection Fenestra LC at the dose of 15 mL/kg. It is possible to distinguish liver from surrounding soft tissues in this image. 

## 5. Conclusion

A prototype cone-beam micro-CT system for small animal imaging is described in this paper. To acquire images with high spatial resolution and low artifacts, data preprocessing and geometry calibration are performed before reconstruction. FDK method is adopted for reconstruction with GPU acceleration. The 3D data postprocessing are performed by the 3DMed software.

Some system characteristics are evaluated by standard phantoms. System spatial resolution is investigated in terms of MTF, which reaches about 14.5 lp/mm at a magnification ratio of 2.36 with Ram-Lak filter. Water phantom study shows that the voxel noise variance is inversely proportional to the exposure intensity. Low contrast resolution is also studied in the paper. Some living mouse imaging results with and without contrast agents are presented to give an overall performance of the system.

For micro-CT, relative low soft tissue contrast resolution is a drawback which hinders its widespread. As is measured in water phantom experiments, the standard deviation is about 35 HU for 50 kVp, 1.2 mA, while CT number of human's main organs, except lung and bone, are mostly in the range from 20 HU to 80 HU [30]. So it is a big challenge to distinguish one from others, especially for the liver. Fortunately, exogenous contrast agent overcomes this problem to some extent. With specific micro-CT contrast agent Fenestra LC, liver is enhanced evidently. Beside contrast agent method, techniques to reduce image noise could also be used in micro-CT to improve contrast resolution. 

At present, several commercial micro-CT systems have been developed [31]. Compared with these systems, one characteristic of our system is reconstruction using GPU hardware, which reaches a high reconstruction speed with relative low cost, and will achieve software upgrade easily. A simple method has been proposed to access geometric calibration results and evaluate the detector horizontal offset utilizing a wire phantom. In addition, as mentioned, the final purpose is to build a BLT-CT dual modality system. A prototype micro-CT system will be more conveniently to integrate with BLT system. Related works have been doing and will be reported in the future.

## Figures and Tables

**Table 1:** Micro-CT scanning protocols for phantoms and mouse.

Protocol	Wire phantom	Water phantom	Mouse
Target voltage	45 kVp	50 kVp	50 kVp
Target current	1.0 mA	0.4, 0.6, 0.8, 1.0, 1.2 mA	1.2 mA
Integrated time	0.6 s/view	0.6 s/view	0.6 s/view
SDD	498 mm	498 mm	498 mm
Magnification ratio	1.3 and 2.36	1.3	1.3
Number of views	500	500	500
Scan mode	360° full scan	360° full scan	360° full scan
Voxel size	0.01 × 0.01 × 0.05 mm^3^	0.1 × 0.1 × 0.1 mm^3^	0.1 × 0.1 × 0.16 mm^3^

**Table 2:** Mean value and standard deviation (SD) in different five ROIs with different exposure intensities (in HU).

	50 kVp, 0.4 mA		50 kVp, 0.6 mA		50 kVp, 0.8 mA		50 kVp, 1.0 mA		50 kVp, 1.2 mA	
	Mean	SD	Mean	SD	Mean	SD	Mean	SD	Mean	SD
Center	−18.9	66.1	−18.0	53.9	−18.7	46.7	−18.4	42.2	−16.1	38.0
Top	4.0	59.3	6.1	47.9	5.3	41.4	4.7	37.6	5.0	33.7
Bottom	−0.2	59.9	−0.4	48.3	0.5	42.2	1.0	37.3	1.9	34.7
Left	−0.6	59.6	−0.4	48.5	0.5	42.6	1.2	37.8	1.3	34.1
Right	5.5	59.3	6.6	47.6	4.9	41.2	4.7	36.9	4.8	33.6
Average difference between center and periphery	16.9 ± 9.8		16.8 ± 10.0		17.3 ± 9.9		17.0 ± 9.7		15.5 ± 8.8	
Average SD	60.8 ± 3.0		49.3 ± 2.6		42.9 ± 2.2		38.4 ± 2.2		34.8 ± 1.8	
